# Genetic diversity and structure of *Saussurea polylepis* (Asteraceae) on continental islands of Korea: Implications for conservation strategies and management

**DOI:** 10.1371/journal.pone.0249752

**Published:** 2021-04-08

**Authors:** Seon A. Yun, Seung-Chul Kim

**Affiliations:** Department of Biological Sciences, Sungkyunkwan University, Suwon, Gyeonggi-do, Korea; University of Massachusetts, UNITED STATES

## Abstract

*Saussurea polylepis* Nakai is an herbaceous perennial endemic to Korea and is highly restricted to several continental islands in the southwestern part of the Korean Peninsula. Given its very narrow geographical distribution, it is more vulnerable to anthropogenic activities and global climate changes than more widely distributed species. Despite the need for comprehensive genetic information for conservation and management, no such population genetic studies of *S*. *polylepis* have been conducted. In this study, genetic diversity and population structure were evaluated for 97 individuals from 5 populations (Gwanmaedo, Gageodo, Hongdo, Heusando, and Uido) using 19 polymorphic microsatellites. The populations were separated by a distance of 20–90 km. We found moderate levels of genetic diversity in *S*. *polylepis* (*Ho* = 0.42, *He* = 0.43). This may be due to long lifespans, outcrossing, and gene flow, despite its narrow range. High levels of gene flow (*Nm* = 1.76, mean *Fst* = 0.09), especially from wind-dispersed seeds, would contribute to low levels of genetic differentiation among populations. However, the small population size and reduced number of individuals in the reproductive phase of *S*. *polylepis* can be a major threat leading to inbreeding depression and genetic diversity loss. Bayesian cluster analysis revealed three significant structures at K = 3, consistent with DAPC and UPGMA. It is thought that sea level rise after the last glacial maximum may have acted as a geographical barrier, limiting the gene flow that would lead to distinct population structures. We proposed the Heuksando population, which is the largest island inhabited by *S*. *polylepis*, as a source population because of its large population size and high genetic diversity. Four management units (Gwanmaedo, Gageodo, Hongdo-Heuksando, and Uido) were suggested for conservation considering population size, genetic diversity, population structure, unique alleles, and geographical location (e.g., proximity).

## Introduction

Population genetic studies often elucidate demographic histories and evolutionary processes in humans [1, 2], animals [3, 4], and plants [5–7]. A few of the many plant studies focus on broad topics, including comparisons of the diversity patterns between wild and cultivated *Sorghum bicolor* [8], identification of introduction events of *Ambrosia artemisiifolia* [7], verification of interspecific gene flow in *Helianthus* [9], and assessment of the genetic diversity and structure of numerous endangered species [10–12]. In particular, genetic diversity and structure, as indicators of gene flow, genetic drift, and differentiation, are essential to understanding the relationships within and among populations.

Islands are an excellent site to study evolutionary processes and to test ecological models (e.g., stepping-stone, source-sink, and metapopulation dynamics) because of their well documented geological ages and geographic isolation [13]. There are two types of islands: oceanic and continental, the latter of which can form from continental shelves or land bridges. Oceanic islands are usually formed by volcanic activity and have never been connected to the mainland [14]. Due to the low frequency of colonization on oceanic islands, biodiversity has largely arisen through the evolution and adaptation of the few initial colonists. Subsequently, the level of endemism is high. [15]. The precise levels of these characteristics are determined by the diversity of ecological habitats and the degree of geographic isolation from the mainland. Many prior studies have focused on patterns of speciation [16–18] and diversification [19] as well as comparisons of genetic diversity between mainland and island populations [20, 21]. On the other hand, continental islands were often created by sea-level change during the Pleistocene glacial and interglacial periods [22]. Novel environmental conditions and small populations isolated by geographical barriers can lead to increased genetic differentiation [23] and new opportunities for allopatric speciation [24].

Korea contains over 1,000 continental islands mostly located in the southern or southwestern regions of the Korean peninsula, and large portions of these islands have been designated as a national park. They harbor numerous plant endemic species, including *Hemerocallis hongdoensis* (Asphodelaceae), *Hosta yingeri* (Asparagaceae), *Potentilla gageodoensis* (Rosaceae), and *Saussurea polylepis* (Asteraceae). Kang and Chung [25] reported low genetic diversity in *Hemerocallis hongdoensis* because of relatively small habitats and restricted geographic distribution. Their result was consistent with previous studies that island populations have lower levels of genetic variation [21] and small population size negatively affects genetic diversity [26]. Several species in islands showed that within-population diversities were significantly high and low levels of genetic differentiation among the populations [22, 27]. Such results explained gene flow influence the differences in genetic diversity and population structure. Although the genetic diversity of some endemic species on the Korean continental islands has been reported [25, 28], it is insufficient to understand the genetic and demographic histories of the endemic species on these continental islands, specifically located in the southwestern part of the Korean peninsula.

The level of genetic diversity is influenced by a variety of factors, including longevity, mating system, and gene flow that is difficult to measure directly. In general, long-lived and outcrossing plant species have higher genetic diversity than short-lived and selfing ones [29]. However, if they are in small or fragmented populations, outcrossing species may have more negative effects on genetic diversity due to depending on distribution, abundance, and the behavior of pollinators or other vectors for dispersal [30]. In addition, in small populations, inbreeding is likely to cause a reduction in the fitness of individuals (especially offspring) due to an increase in homozygosity and therefore the expression of more deleterious recessive alleles [31]. Angeloni et al. [32] highlighted how population size influenced the magnitude of inbreeding depression in plants. Interestingly, their result showed that inbreeding is a common phenomenon in plant and inbreeding depression significantly increases with population size. Nevertheless, theoretical predictions and empirical evidence have suggested that inbreeding in small population result in the expression of deleterious recessive alleles, low genetic diversity and fitness [31–33]. This, in turn, can cause a further reduction in population size, making the population less capable of sustaining itself. Self-incompatibility (SI) is the most widespread strategy to promote outcrossing and increase genetic diversity in flowering plants [34]. However, this system may not be as effective at establishing new populations or restoring the size of small or fragmented populations due to its inherent mating restrictions [35]. Moreover, the reduction of population size in self-incompatible species can result in the loss of the self-incompatibility locus (S-locus) [36], and subsequently a further loss of genetic diversity and fitness. Therefore, maintenance of constant population size should be considered one of the most fundamental factors protecting species from threats such as inbreeding depression and loss of genetic diversity. To achieve these ends, we can consider two processes: *in situ* and *ex situ* conservation. In many cases, *in situ* conservation may be impractical or impossible without eliminating threats such as climate change, habitat destruction, and other anthropogenic activities. Conversely, *ex situ* conservation through seed and germplasm storage is an important method for preserving species for augmentation/reintroduction when environmental pressures are released and suitable locations become available for reintroduction [37]. Augmentation/reintroduction can increase population size, thereby leading to a self-sustaining population, and insulating species from threats such as loss of genetic diversity.

*Sasssurea polylepis* as an endemic species is highly restricted to mountains in fewer than 10 continental islands located off the southwestern coast of the Korean peninsula and it is the only *Saussurea* species found there. As with *S*. *esthonica* [38], *S*. *gnaphalodes* [39], *S*. *chabyoungsanica* [40], and *S*. *involucrata* [41], the populations are small due to climatic changes and recent/ongoing habitat destruction by humans and herbivores. Therefore, *S*. *polylepis* is currently categorized as vulnerable species [VU B2ab (iii, iv)] in the Korean Red List [42]. In addition, *S*. *polylepis* is edible, so it is very likely that the population size will decrease more rapidly by humans, such as *S*. *lapa*, a medicinal plant [43]. Despite the need for conservation of endemic species, there is no study of *S*. *polylepis*. Thus, the aims of this study were to (1) assess the genetic diversity and population genetic structure of *S*. *polylepis* using microsatellite markers, and (2) develop and implement the conservation strategies based on genetic diversity. This study will improve our understanding of the demographic and evolutionary history of endemic species on the continental islands and provide fundamental baseline knowledge for developing and implimenting conservation strategies.

## Materials and methods

### Plant materials

**Fig 1:**
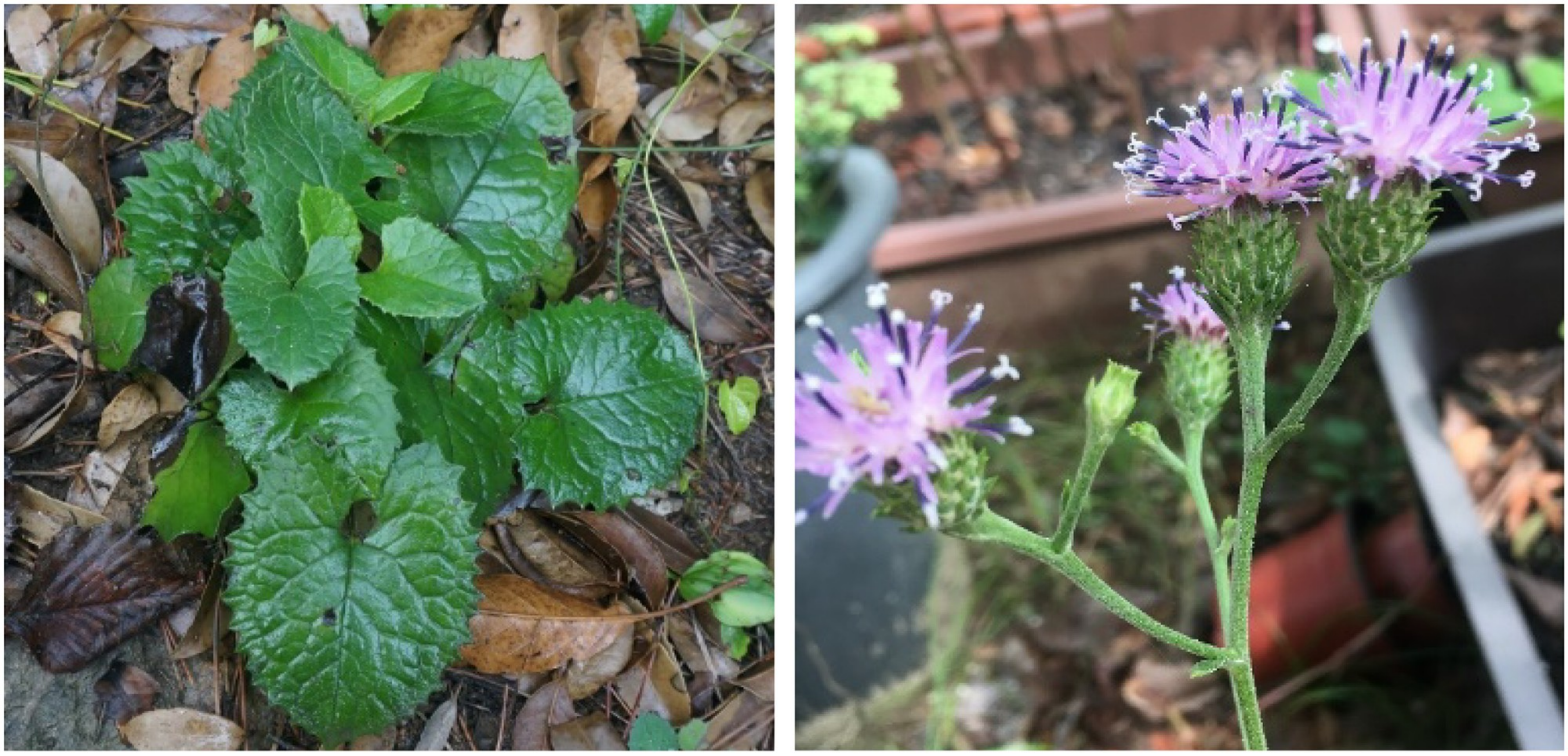
Photographs of *Saussurea polylepis* in Heuksando. Found only on 6 southwestern continental islands of Korea and mainly observed around hiking trails. Climate change, anthropogenic activities, and herbivores are the main threats to population decline.

**Fig 2:**
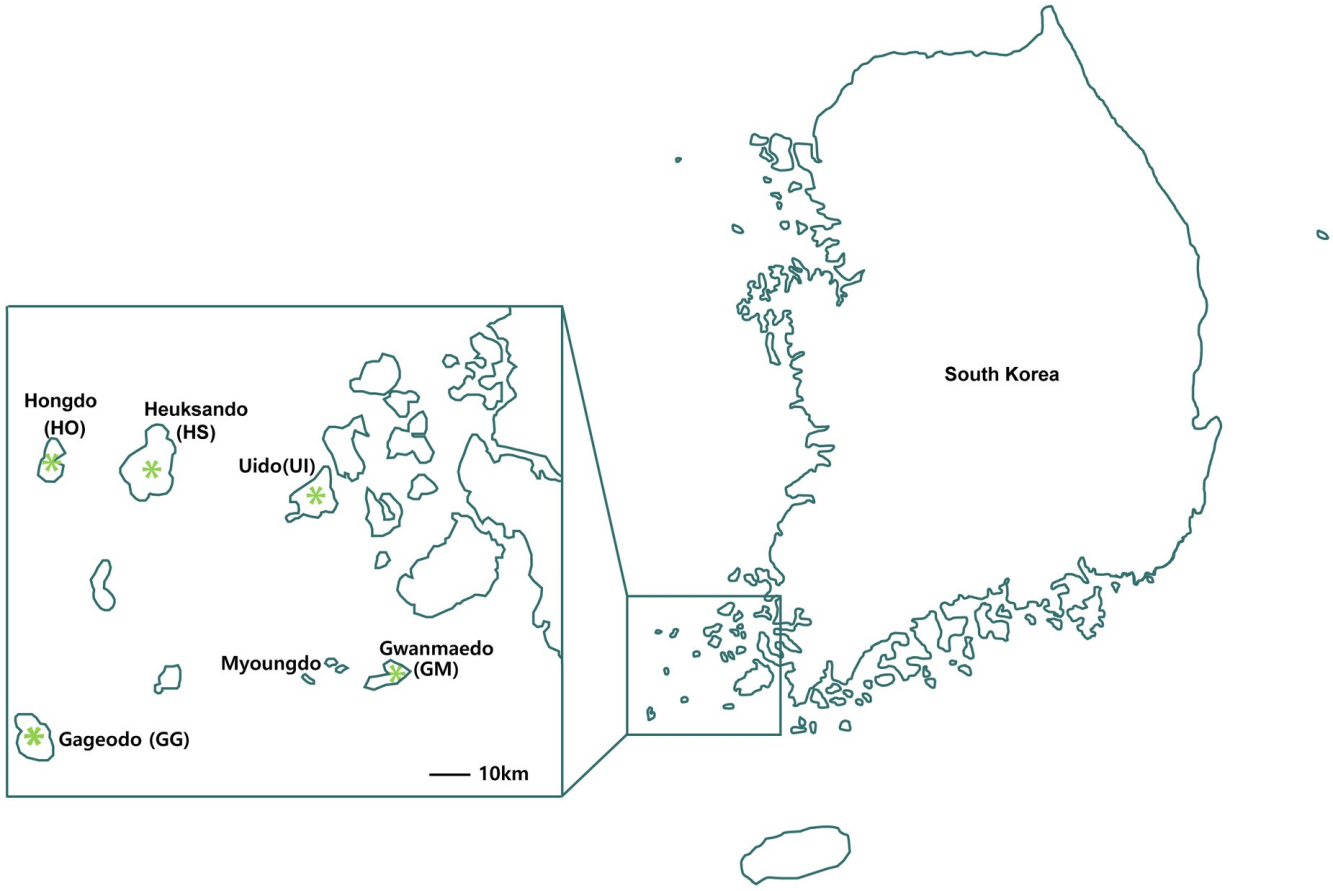
Distribution map of *Saussurea polylepis*. Collection sites are marked with the asterisk. The source of the original map image: SEDAC (Socioeconomic Data and Applications Center), http://www.worldofmaps.net/, accessed November 21, 2020.

The perennial herb *Saussurea polylepis* Nakai (Fig 1) is distinctive among congeneric species by the presence of glossy and reniform basal leaves, hairs on adaxial and abaxial leaf surfaces, petioles without wings, and irregularly dentate leaf margins [44]. In a preliminary phylogenetic analysis based on the combined nuclear ribosomal DNA ITS (internal transcribed spacer) and ETS (external transcribed spacer) dataset (Yun S. A., unpublished data), *S*. *polylepis* formed a monophyletic group very closely related to *S*. *neoserrata*, which has elliptic or linear leaves with winged petiole and numerous capitula in a corymbiform inflorescence. In terms of distribution, *S*. *neoserrata* is widely distributed inland of Korea, Mongolia, and Russia, whereas *S*. *polylepis* is highly restricted to southwestern continental islands (Fig 2). Given the phylogenetic relationship and distribution pattern, it is conceivable that *S*. *polylepis* may have originated from *S*. *neoserrata* through allopatric speciation (i.e., peripheral isolation model).

Distribution information for sampling was accessed and confirmed through literature and specimens from the Korea National Arboretum (Korea Forest Service) and National Institute of Biological Resources (Ministry of Environment). A total of 97 individuals of *S*. *polylepis* were sampled to analyze the genetic diversity and population genetic structure from five continental islands: Gwanmaedo (GM), Gageodo (GG), Hongdo (HO), Heuksando (HS), and Uido (UI). Although *S*. *polylepis* also occurs on Myoungdo located approximately 15 km west of GM (Fig 2), it was excluded from this study due to inaccessibility. In each population, fresh leaves were collected from 2 to 50 individuals after estimating the relative population size and the percentage of individuals collected from total observed individuals ranged from 20% to 50% (Table 1). Of the five islands where *S*. *polylepis* was collected, GM is the smallest (5.73 km^2^). Since less than 10 individuals were observed and most were seedlings with a single leaf, only two individuals were collected. Although a greater number of samples are needed for more robust conclusions, additional individuals in GM could not be found over a 3-year survey period (2017–2019; Yun S. A., unpublished data). GG is an island located at the far southwestern Korean peninsula and has an area of 9.18 km^2^. Approximately 50 individuals were found, but most of them were very young, allowing only 17 mature individuals to be collected. HO is located about 20 km west of HS and has an area of 6.47 km^2^. Thirty-three individuals were observed along the trail, and as with GM most were young. This allowed the collection of a total of 13 individuals. In HS, which has a 21.7 km^2^ area, *S*. *polylepis* was continuously distributed along the trail to the summit. A total of 50 individuals were collected, which represented more than 40% of the total observed individuals. UI is the second largest (10.7 km^2^) of the five islands, and of 30 individuals observed, we collected a total of 15 mature individuals. Therefore, the total number of individuals sampled in this study from five islands (i.e., 97 individuals) represents reasonably good proportion of actual species distribution range and population size on each island. All samples were collected with permission issued from the Korea National Park Service and voucher specimens for each population were deposited in the Ha Eun Herbarium, Sungkyunkwan University (SKK). We thank the Korea National Park Service to permit sample collection of *Saussurea polylepis* from five islands.

**Table 1:** Locality and voucher information of five *Saussurea polylepis* populations used in this study.

Collection locality (abbreviation)	Latitude (N)	Longitude (E)	Island size (km^2^)	N (%)^a^	Voucher specimen
Gwanmaedo, Jeollado, Korea (GM)	34.235487	126.047873	5.73	2 (20)	-
Gageodo, Jeollado, Korea (GG)	34.075722	125.108583	9.18	17 (34)	-
Hongdo, Jeollado, Korea (HO)	34.696222	125.201944	6.47	13 (40)	SKK044834
Heuksando, Jeollado, Korea (HS)	34.676056	125.426361	21.7	50 (40)	SKK044835
Uido, Jeollado, Korea (UI)	34.599333	125.814972	10.7	15 (50)	SKK044836

Voucher specimens were deposited in the Ha Eun Herbarium, Sungkyunkwan University (SKK).

^a^ Percentage of individuals collected from total observed individuals.

### DNA extraction, PCR amplification and genotyping

The total genomic DNA was extracted using the DNeasy Plant Mini Kit (Qiagen, Carlsbad, California, USA), following the instructions of the manufacturer. PCR was performed using 19 SSR primers (S1 Table) as previously developed [45]. The PCR was conducted with a final reaction volume of 20μL containing 1μL of genomic template DNA, 0.5μL of 10 pmol forward primer with fluorescence dye (HEX or FAM), 0.5μL of 10 pmol reverse primer, 2.5μL (with 2.5mM MgCl_2_) of PCR buffer, 0.5μL (each 10mM) of dNTPs, 0.1μL (5U/μL) of *Taq* DNA polymerase (Inclone Biotech, Gyeonggido, Korea), and 14.9μL of distilled water. The PCR program consisted of initial denaturation at 95°C for 1 min, followed by 35 cycles at 95°C for 20 s, annealing at 58–60°C for 40 s, extension at 72°C for 60 s, and a final extension at 72°C for 5 min. The PCR amplicons were then detected using an ABI 3730XL DNA Sequencer (Applied Biosystems, California, USA) at the Macrogen company (Seoul, Korea), and allele sizes were determined by comparing with a GeneScan 500 LIZ Size Standard using the software GeneMapper version 5.0 (Applied Biosystems).

### Data analysis

The presence of null alleles was investigated using Microchecker 2.2.3 [46], with a confidence interval level of 0.05. Null allele frequencies across the populations were estimated using expectation maximization (EM) in the program FreeNA [47] with 1,000 bootstrap resamples, to avoid bias in the population structure analysis. The uncorrected global *Fst* was additionally compared to *Fst* values corrected using the excluding null alleles (ENA) method.

Genetic diversity parameters, including mean number of alleles (*Na*), number of effective alleles (*Ne*), observed heterozygosity (*Ho*), expected heterozygosity (*He*), inbreeding coefficient (*F*), and proportion of polymorphic loci (P%), were estimated using GenAlEx v.6.5 [48]. Allelic richness was estimated in FSTAT v2.9.4 [49]. Departures from Hardy-Weinberg equilibrium (HWE) and linkage disequilibrium (LD) for each pair of loci in each population were tested using Genepop 4.2 [50] with 10,000 dememorizations and in 1,000 batches with 10,000 iterations per batch. GenAlEx v.6.5 [48] was also used to estimate the genetic differentiation coefficient (*Fst*) and number of migrants (*Nm*).

An analysis of molecular variance (AMOVA) was conducted using Arlequin version 3.5 [51] to estimate the genetic variance at different hierarchical levels: (1) two hierarchical levels (among and within populations) and (2) three hierarchical levels (among regions, among populations within regions, and within populations). The significance of the variance components was estimated using 1,023 permutations. Population genetic relationships were examined using discriminant analysis of principal components (DAPC) with the R package *adegenet* [52]. An UPGMA (unweighted pair group method of arithmetic averages) was conducted to elucidate the relationships among populations based on Nei’s genetic distance matrix using MEGA-X [53], and the statistical support for branches was calculated using 1000 replicates in PAST version 2.17 [54].

Bayesian model-based clustering was applied by STRUCTURE v.2.3.4 [55] to characterize the population structure. An admixture model was used to estimate the number of population clusters (K) ranging from one to ten. Each STRUCTURE run was performed with 1 x 10^5^ burn-ins and 1 x 10^5^ MCMC iterations with 20 runs per K value. Best K was identified based on the approach of Evanno et al. [55] by STRUCTURE HARVESTER v.0.6.94 [56]. The graphical result was displayed using CLUMPAK [57].

To detect evidence of recent bottlenecks in the populations, we tested the mode-shift based on allele frequency using BOTTLENECK v.1.2.02 software [58]. Wilcoxon signed-rank tests were also conducted to verify the presence of an excess of heterozygosity with assumptions for two mutation models: step-wise mutation model (SMM), and a two-phase model (TPM). The proportion of SMM in TPM was set to 70%.

## Results

### Population genetic diversity

In this study, 19 polymorphic microsatellite markers were used to determine the genetic diversity of *S*. *polylepis*. Null alleles were detected in all but one population (i.e., GM) (Table 2), and four populations commonly had SP3 and SP13 loci with null alleles. As there was little difference between the corrected and uncorrected *Fst* values (corrected *Fst* = 0.083, 95% confidence interval (CI): 0.063–0.108, uncorrected *Fst* = 0.084, 95% CI: 0.066–0.108) (S2 Table), the original data set was used for the analysis. Genetic indices of each population were summarized in Table 2. *Na* ranged from 1.47 (GM) to 6.32 (HS) across 19 loci, and *Ne* varied from 1.48 (GM) to 2.87 (HS). The average *Ho* value was 0.42, ranging from 0.35 (HO) to 0.47 (GM). The *He* value of GM was the lowest, whereas that of GG was the highest. Excluding GM, the four populations all had *He* values higher than *Ho* values, indicating heterozygote deficiency. Inbreeding coefficient ranged from -1.00 in GM to 0.20 in HS (Table 2). On average, the percentage of polymorphic loci across all populations was 84.21%, and HS had the highest degree of polymorphism (100%), followed by GG and UI with 94.74% (Table 2). Allelic richness was estimated using two data sets, with and without GM. The results including GM showed that GG had the highest value of 2.20 and GM had the lowest value of 1.47. When allelic richness was estimated excluding GM, HS had the highest value of 4.45 and HO had the lowest value of 3.63 (Table 2). Private alleles were observed in all populations (Table 2), and the most private alleles were observed on HS, having 21. The average gene flow value (*Nm*) across five populations was 1.76.

**Table 2:** Genetic diversity statistics over 19 loci for five populations of *Saussurea polylepis*.

Regions	N	*Na*	*Ne*	*Ho*	*He*	*F*	P (%)	Allelic richness (excluding GM)	No. of private alleles	Locus with null allele
GM	2	1.47	1.47	0.47	0.24	-1.00	47.37	1.47 (-)	3	NA
GG	17	4.47	2.76	0.46	0.52	0.13	94.74	2.20 (4.22)	10	SP3, SP13, SP26
HS	50	6.32	2.87	0.39	0.49	0.20	100	2.15 (4.45)	21	SP3, SP12, SP13, SP22, SP23, SP26, SP34, SP35
HO	13	3.63	2.40	0.35	0.44	0.14	84.21	2.00 (3.63)	9	SP3, SP13, SP23, SP35
UI	15	4.42	2.52	0.44	0.49	0.09	94.74	2.13 (4.26)	14	SP3, SP13, SP34
Mean		4.06	2.40	0.42	0.43	0.01	84.21	1.99 (4.14)	11.4	

N = Number of individuals, *Na* = Number of different alleles, *Ne* = Number of effective alleles, *Ho* = Observed heterozygosity, *He* = Expected heterozygosity, *F* = Fixation index (inbreeding coefficient), P = Percentage of polymorphic loci. NA = not applicable.

### Population structure and genetic relationships

**Fig 3:**
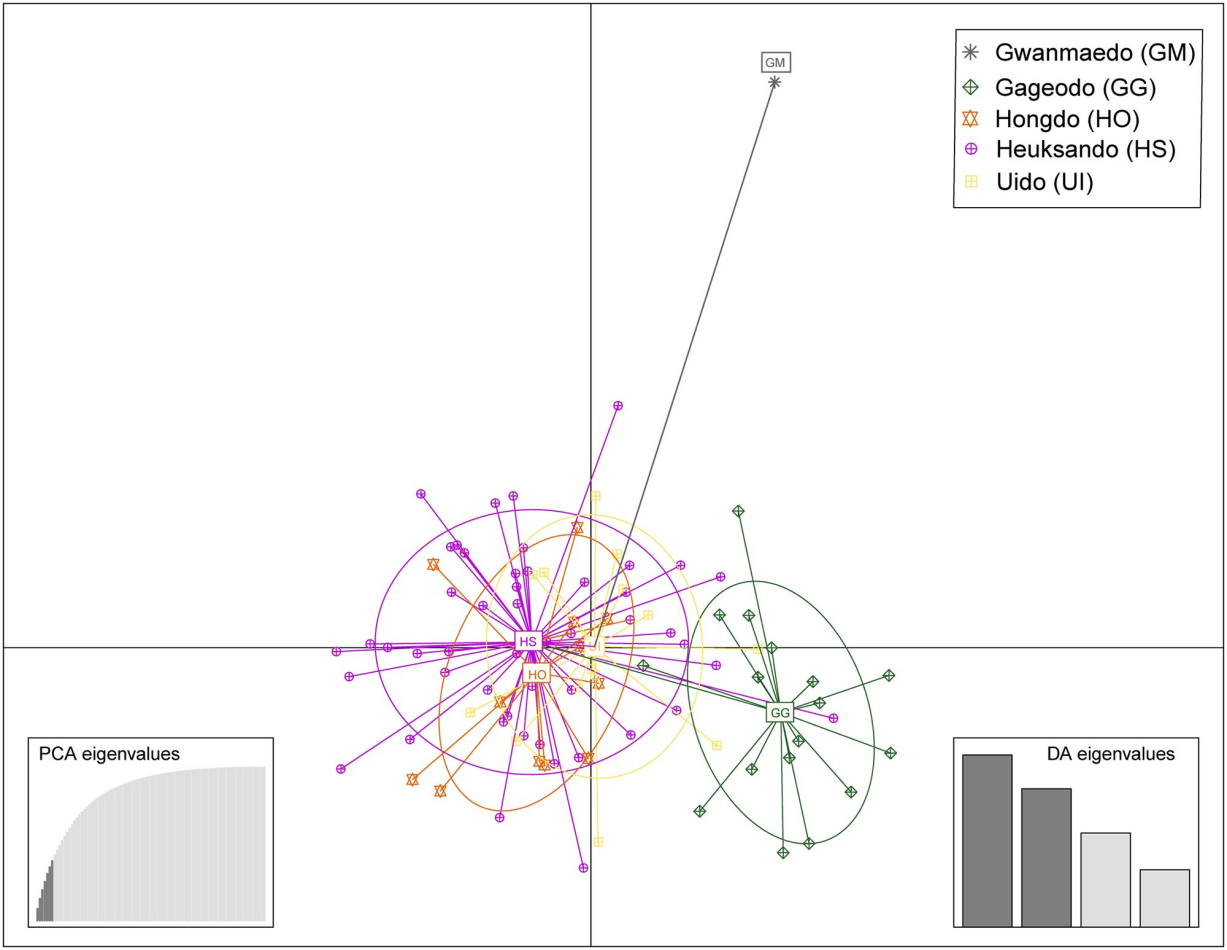
Scatterplot output from DAPC for the genetic structure of *Saussurea polylepis* individuals. Dots represent individuals from the 5 populations, and each color and symbol represent different populations.

**Fig 4:**
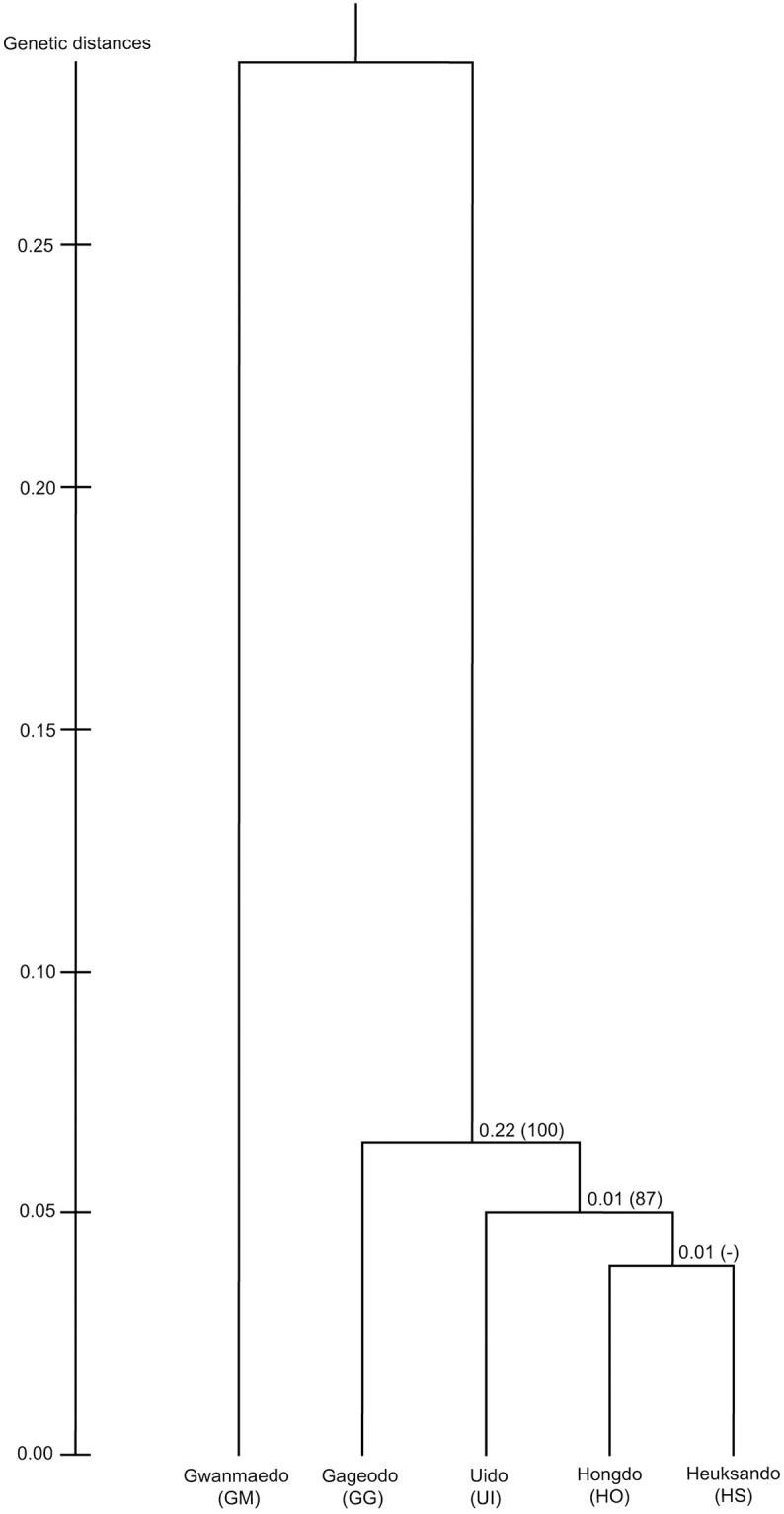
UPGMA dendrogram based on Nei’s genetic distance. Numbers above branches are indicated genetic distances. Bootstrap values are shown in parentheses, and hyphen indicates a less than 50% bootstrap value.

**Fig 5:**
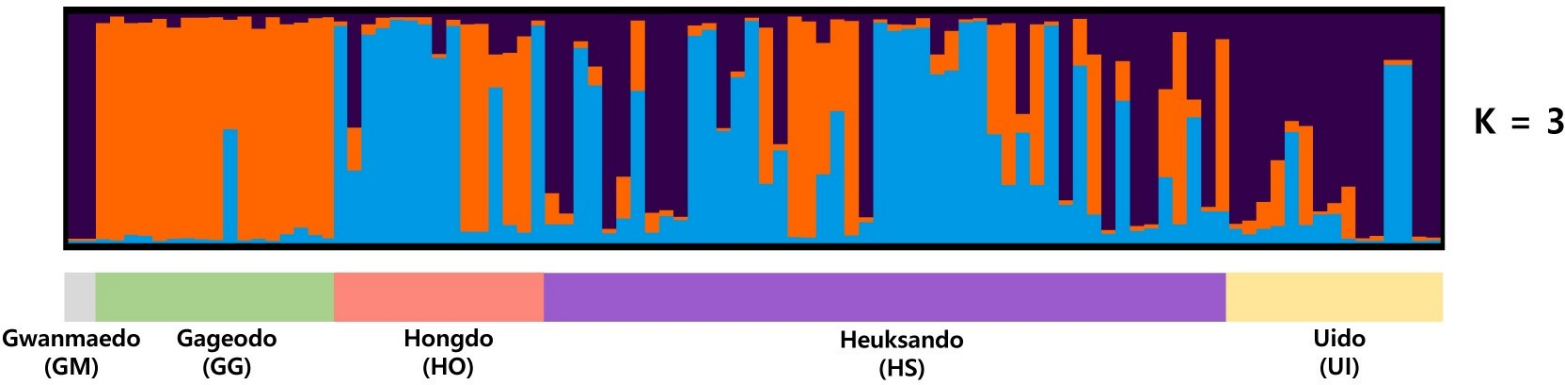
STRUCTURE bar plot of 97 individuals of *Saussurea polylepis* from five populations at K = 3. Color indicates different clusters.

The AMOVA analysis with all populations set as one group (Table 3) revealed that the genetic variation within the population (91.04%) is higher than variation among populations (8.96%). The result of AMOVA using three groups (GM, GG, and HO-HS-UI) defined by UPGMA and DAPC analyses revealed the most genetic variation was attributed to differences within populations (88.23%). Genetic differences among populations and among populations within groups respectively accounted for 6.02% and 5.75% of genetic variation (Table 3). As shown in Fig 3, *S*. *polylepis* individuals formed three groups according to DAPC analysis. The scatterplot shows that most of the individuals from HO, HS, and UI populations were overlapped and HS consisted of highly variable individuals and widely positioned. The UPGMA result (Fig 4) showed three groups (GM, GG, and HO-HS-UI), of which GM was the most divergent, while the genetic distance between HS and HO was the closest with genetic distance of 0.08 (S3 Table). The Bayesian clustering analysis showed that the optimal number of genetic clusters for which ΔK reached a maximum value was 3 (S1 Fig). Four populations, GG, HO, HS, and UI, were identified as an admixture of three genetic clusters (Fig 5). Based on non-admixture structure of GM and different admixture patterns, there were three groups, GM, GG, and HO-HS-UI, with specific genetic structures at K = 3. It was consistent with the results of UPGMA and DAPC. Genetic differentiation based on F-statistics (*Fst*) showed that HO-HS had the lowest value (0.03), while GM-HO had the highest (0.31), indicating significant genetic differentiation (S3 Table).

**Table 3:** AMOVA results for 97 *Saussurea polylepis* individuals among and within populations.

Source of variation	d. f.	Sum of squares	Variance components	Percentage of variation	Fixation indices	*p*-value
Among populations	4	78.45	0.46	8.96	*Fst* = 0.09	0.00 ± 0.00
Within populations	189	891.94	4.72	91.04		
Total	193	970.39	5.18			
Three groups (GM, GG, and HO-HS-UI)						
Among groups	2	43.87	0.32	6.02	*Fct* = 0.06	0.10 ± 0.01
Among populations within groups	2	34.58	0.31	5.75	*Fsc* = 0.06	0.00 ± 0.00
Within populations	189	891.94	4.72	88.23	*Fst* = 0.12	0.00 ± 0.00
Total	193	970.39	5.35			

The bottleneck analysis was conducted to identify excesses or deficits of heterozygosity related to population expansions or bottlenecks. Although the mode-shift tests detected evidence of a bottleneck only in GM (Table 4), we cannot accurately determine the presence or absence of a bottleneck or population expansion because a minimum of 10 individuals is recommended for bottleneck analysis. The distribution of alleles in GG, HO, HS, and UI populations showed a normal L-shape, which demonstrates the absence of a recent bottleneck or expansion. Lastly, no excess of heterozygosity was detected in any of the 4 populations: GG, HO, HS, and UI (Table 4).

**Table 4:** The results of Wilcoxon’s signed-rank test and mode shift test implemented in BOTTLENECK software.

Populations	Wilcoxon’s test		Mode shift
	TPM	SMM	
GM	0.00	0.00	Shift
GG	0.32	0.91	Normal L-shaped
HS	0.99	1.00	Normal L-shaped
HO	0.12	0.55	Normal L-shaped
UI	0.96	1.00	Normal L-shaped

Wilcoxon’s signed-rank tests were conducted under the two-phase models (TPM) and stepwise mutation models (SMM), and the values indicated the probability for one tailed heterozygote excess. Normal L-shape revealed that populations had not undergone a recent bottleneck event.

## Discussion

### Moderate level of genetic diversity

High genetic diversity is important because it can allow populations to more easily adapt to environmental changes. Therefore, understanding the level of genetic diversity within and among populations is essential to establish conservation strategies of species [31]. In the present study, the genetic diversity of *S*. *polylepis* was investigated. Based on 19 SSR loci, levels of genetic diversity within the population (except for GM) as well as mean heterozygosity values were similar or higher (Table 2) when compared with heterozygosity values (mean *He* = 0.42 estimated by using microsatellite DNA data) of endemic species reported by Nybom [29]. In addition, *S*. *polylepis* had similar values to *Hypochaeris catharinensis* of Asteraceae (*He* = 0.44) [59], which had moderate levels of genetic diversity. On the other hand, *S*. *polylepis* had relatively low genetic diversity compared to endemics *Michelia coriacea* (*He* = 0.470) [60] and *Antirrhinum charidemi* (*He* = 0.48) [61], demonstrated high genetic diversity. Given these comparisons, the diversity of *S*. *polylepis* can be assessed as relatively moderate.

The genetic diversity of plant species is related to distribution range, population size, life cycle, mating system, and gene flow [62]. Of these factors, a narrow distribution range and small population size tend to decrease genetic diversity [31]. In particular, as inbreeding generally exposes recessive deleterious alleles by increasing genetic homozygosity, inbreeding depression is considered a major threat to small and isolated populations [63]. In addition, inbreeding increases departure from HWE and linkage disequilibrium (LD) [64], and diversity reduction in inbreeding populations resulting in high homozygosity reduces the effectiveness of recombination throughout the genome [65]. However, selection can decrease recessive alleles in a population through a process referred to as purging, thus reducing future inbreeding depression [66]. Although purging can reduce the level of inbreeding depression as well as fitness decline during inbreeding periods [67, 68], inbreeding depression can nonetheless strongly affect genetic diversity in the long term [65]. Considering that small populations and departure from HWE (S2 Table) and LD (S4 Table) were observed in *S*. *polylepis*, the influence of inbreeding on the genetic diversity of *S*. *polylepis* still cannot be overlooked and further analyses about inbreeding are needed to get accurate conclusion.

The moderate genetic diversity of *S*. *polylepis* can be explained by the perennial life history, mating system, and gene flow. Although the primary pollinator remains unclear, bees and flies were seen to be involved in the pollination of *S*. *polylepis* as with *S*. *weberi* [69], *S*. *laniceps*, and *S*. *medusa* [70]. Asteraceae is one of the families with a self-incompatibility system, which is controlled by a single S-locus with multiple S-alleles [71]. Although *S*. *obvallata* within *Saussurea* was reported as a self-compatible species [72], the fact that self-pollination could not produce mature fruit directly supports self-incompatibility of *S*. *polylepis*. Young and Pickup [73] demonstrated that small populations have low S-allelic diversity and exhibit a significant reduction in seed sets relative to large populations with a higher number of S-alleles. Their result is consistent with research conducted by Reinartz and Les [74], which found that seed set was limited by a low number of S-alleles in *Aster furcatus*. The outcrossing and self-incompatibility system are important means for increasing genetic diversity and SI species tend to have higher diversity. However, the reduced genetic diversity including S-alleles due to small populations and bottlenecks (or founder effect) of *S*. *polylepis* can lead the lack of reproductive phase individuals (mates) and reduction of offspring density due to low seed set can cause the slow recovery of population size and subsequently increase extinction risk. Therefore, the reliance on outcrossing and self-incompatibility in *S*. *polylepis* within small populations can be obstacles to increasing or maintaining genetic diversity due to a lack of pollinators and other nearby reproductive phase individuals.

The degree of gene flow can be confirmed from the AMOVA (Table 3). AMOVA results indicated that most genetic variation can be attributed to differences within populations, while genetic differences among populations accounted for a smaller portion of genetic variation. The average gene flow value (*Nm* = 1.76) across five populations can also support gene flow.

GM showed higher *Ho* value than *He* value. The *Ho* value was less than the *He* value in GG, HO, HS, and UI, indicating a deficiency of heterozygotes. The decreased *Ho* value might be explained by overlapping generation [75], inbreeding, assortative mating, the Wahlund effect, and the presence of null alleles [28, 76]. Genetic diversity typically declines faster in species with overlapping generations [75, 76]. Because *S*. *polylepis* is likely insect-pollinated and has self-incompatibility, assortative mating may not adequately explain heterozygote deficiency. The Wahlund effect may provide a better explanation, as it can be caused by the sampling of individuals from two or more subpopulations with different genotypic frequencies within a population presumed to be a single population. Also, the presence of null alleles in four populations may have led to an underestimation of observed heterozygosity.

HS was the largest population and had the most private alleles, in accordance with the results of previous studies, suggesting that genetic diversity is positively correlated to population size [31, 77]. This can subsequently relate to source-sink population theory, which explains population dynamics in spatially heterogeneous landscapes. Gene flow via seed or pollen dispersal plays a key role in revealing source-sink relationships, and sink habitats are mainly dependent on immigration from source habitats [78]. The size of the source population can be important for determining the extent of gene flow into the sink population, as a larger population can likely provide more pollen and seed dispersal than smaller populations, while smaller populations are more likely to receive genetic sources at a higher rate from larger populations than from other small ones [31]. Also, the classic source-sink population model predicts lower genetic diversity in sink populations than in source populations [10]. Based on this hypothesis, it is conceivable that due to its comparatively large size, HS may serve as a source population characterized by higher genetic diversity (Table 2).

### Genetic differentiation and population genetic structure

In this study, we found substantial genetic diversity within, rather than among populations in *S*. *polylepis* (Table 3). This pattern of genetic structure was also observed in the related species *S*. *involucrata* [41]. Gene flow strongly influences the genetic structure and genetic differentiation of populations [5]. In this study, the degree of gene flow (*Nm*) was indirectly estimated based on *Fst* values across all populations for each locus. Wright [79] showed that when gene flow is *Nm* > 1.0, genetic differentiation may be restricted. This is true for neutral genetic variation and selection can affect to either increase or decrease *Fst* [80]. In addition, microsatellites can be linked to loci subjected to selection. However, microsatellites are primarily neutral, so an *Nm* value of 1.76 obtained from the *Fst* values across all populations of *S*. *polylepis* can be explained the gene flow and low levels of differentiation among populations.

Result generated from the DAPC showed three clear distinctions between populations (Fig 3), especially indicating active gene flow among HO, HS, and UI populations. Gene flow facilitates the influx of new alleles through pollen and seeds. This makes it essential to genetic diversity, as well as the dynamics and maintenance of populations [81–83]. The range of pollen dispersal is directly influenced by the pollination vector (e.g., water, wind, and animals) and environmental conditions (e.g., wind velocity, temperature, etc.). Understanding the effects of these factors on dispersal range has therefore become an important subject of research. For example, Jha and Dick [84] verified that native bees mediate long-distance pollen dispersal (> 1,800 m) on coffee farms, and Pasquet et al. [85] found that *Xylocopa flavorufa* (carpenter bees) visited wild and domesticated populations at a distance of up to 6 km. The shortest distances in this study was approximately 20 km between the HO and HS populations. Therefore, there is most likely a low probability of gene flow by an insect pollinator. On the other hand, the seed of *S*. *polylepis* has pappus, which enable seed dispersal by wind. Long-distance dispersal of seeds by wind has been reported [86, 87], and model-based studies have suggested that seed weight and wind strength play essential roles in the flight time and distance of seed dispersal [81, 88, 89]. Thus, seed dispersal seems to play a more relevant role than pollination regarding gene flow in *S*. *polylepis*.

The pairwise *Fst* values ranged from 0.03 to 0.31 (S3 Table). GM and HO had the highest level of genetic differentiation. However, only two individuals in GM were included. Therefore, any conclusions about genetic diversity should be viewed with caution. Their geographic isolation from each other (ca. 90km) is a likely cause of the observed low gene flow. The pairwise *Fst* value of GM and UI was also high (0.24). Considering that UI is about 40 km from GM, the barriers to seed dispersal are likely not only pure geographic distance, but also factors such as sea currents and wind direction. Excluding GM, genetic differentiation among the other four populations was low (*Fst* = 0.03–0.06). The *Fst* value between HO and HS was the lowest, suggesting that the closer the distance, the more active the gene flow. This is also consistent with the DAPC, UPGMA and STRUCTURE results. In particular, even thought *Nm* and AMOVA results suggested active gene flow and low levels of differentiation, GM and GG had different population structure compared to HO-HS-UI based on DAPC, UPGMA, and STRUCTURE analyses. These differences may be explained by the time of gene flow. The continental islands across which *S*. *polylepis* is distributed are contained within the Yellow Sea. The Yellow Sea is located between the Korean peninsula and mainland China, and was exposed during the Last Glacial Maximum (LGM, 18,000–20,000 years ago) of the Pleistocene when the sea level was 85-140m lower [90, 91]. Sea level fluctuations caused by global climate oscillations during the Pleistocene greatly impacted on the distributions of numerous species through population fragmentation and migration [91–93]. In this case, the LGM land-bridge connected these Yellow Sea islands to each other, which likely increased seed and pollen dispersal. This is thought to have facilitated migration events from HS to HO, UI, GM and GG or among populations during the LGM. However, sea level rise after the LGM segregated these continental islands, limiting gene flow and influencing populations differentiation with these newly arisen geographic barriers. HO has an exceptionally similar genetic structure to HS. It is therefore surmised that these two populations were historically connected when sea-levels were low, and that some amount of gene flow still occurs due to geographic proximity. Future research based on cpDNA is needed for an even more rigorous understanding of the genetic structure relationships and demographic histories between these populations.

### Conservation implications

It is necessary to first establish demographically independent management units in order to maintain adequate population size, especially of endemic or rare species. Based on genetic diversity, genetic differentiation, and geographical distances, GM, GG, HS-HO, and UI may indeed be acceptable management units. GM is composed of a genetic structure (dark blue) shared by the other populations, especially UI (Fig 5), it is nonetheless treated as a management unit because of its private alleles and dangerously small populations size (Table 2). Since GG has a different genetic structure compared to HO-HS-UI, it should be considered individually. HO and HS are geographically close (ca. 20 km) and have similar genetic structures (Fig 5), they should be grouped as a single management unit. Although UI share population genetic structure with HO and HS, considering geographical location from HO or HS, level of habitat disturbance, and unique alleles, it would be appropriate to separate from HO-HS unit.

Increasing the population size of GM should be a priority. Frankham et al. [94] suggested the effective population size (*Ne*) for preventing inbreeding depression and having evolutionary potential. Based on their study, establishing a conservation strategy to retain more than *Ne* ≥ 100 as an initial step should be prioritized. The current small size of GM presents challenges to locally obtaining heterozygous seeds or seedlings, especially considering mate limitation, will therefore inevitably require transplanting or reintroducing nursery-grown *S*. *polylepis*. In addition, strategies for restoring genetic diversity of GM through artificial gene flow may be considered. As GM shared the most genetic structure with UI, we could even set up UI and GM as a conservation unit for artificial gene flow. Based on population size, genetic diversity, and private allele abundance, the HO-HS unit is indisputably the most critically in need of management. Given that gene flow exists between HO and HS, rescue effects may play a role in buffering these populations against extinction [95]. However effective population size of HO should be maintained in order to sustain mate availability and to reduce the extinction risk from small population size. A conservation strategy for HS is needed to maintain the current population level; however, as habitat disturbance from humans and goat predation was observed in both HO and HS, strategies for habitat management should also be developed. Also, securing seeds with divergent alleles, including private alleles, may constitute a suitable component of ex situ conservation. In particular, many orthodox seeds, including those of *Saussurea*, are resistant to water stress and easy to store, so that genetic diversity can be preserved for relatively long periods at low temperatures [96]. In *Saussurea*, it was reported that seeds can be stored at -20 °C [97]. GG and UI have unique genetic structures and private alleles, and there are fewer threats from anthropogenic activities (e.g., tourists) than on the other three island populations. However, the effective population size should be preserved considering the small population size, small number of reproductive phase individuals, risk for inbreeding depression, and loss of heterozygosity. For the persistence of each population, seeds can be an important source of augmentation/reintroduction. Therefore, securing seeds and plant germplasm in seed banks should be given priority, and these can use as sources for population reintroduction. If it is difficult to obtain seeds from a certain populations, seeds or seedlings from other populations can be considered. Unfortunately, the success of reintroductions is low [98] and an initial loss of genetic diversity may hinder future conservation [99]. Therefore, genetic monitoring of current populations should be performed in parallel with *ex situ* strategies [99].

## Supporting information

S1 Fig ΔK values based on the Bayesian analysis from STRUCTURE HARVESTER program. (TIF)

S1 Table Characteristics of 19 microsatellite markers used in this study. (DOCX)

S2 Table Genetic characters of the 19 microsatellite loci. *Na* = No. of different alleles, *Ne* = No. of effective alleles, *Ho* = Observed heterozygosity, *He* = Expected heterozygosity, *F* = Fixation index, *Fst*^*a*^ = global *Fst* without using the ENA correction, *Fst*^*b*^ = global *Fst* using the ENA correction, * Significant departures from Hardy-Weinberg equilibrium at *p* < 0.05.
(DOCX)

S3 Table Pairwise genetic differentiation index (*Fst*) (below the diagonal) and Nei’s genetic distance (above the diagonal) among the five populations. (DOCX)

S4 Table Linkage disequilibrium among 19 primer pairs of five populations. Note: + = significant linkage disequilibrium (*p* < 0.05); − = non-significant linkage disequilibrium; * = unavailable data.
(DOCX)
