# Supplementary figures and images for: Genetic diversity and structure of Saussurea polylepis (Asteraceae) on continental islands of Korea: Implications for conservation strategies and management
Source: PLoS One. 2021 Apr 8;16(4):e0249752. doi: 10.1371/journal.pone.0249752 (PMC8031399; doi:10.1371/journal.pone.0249752)

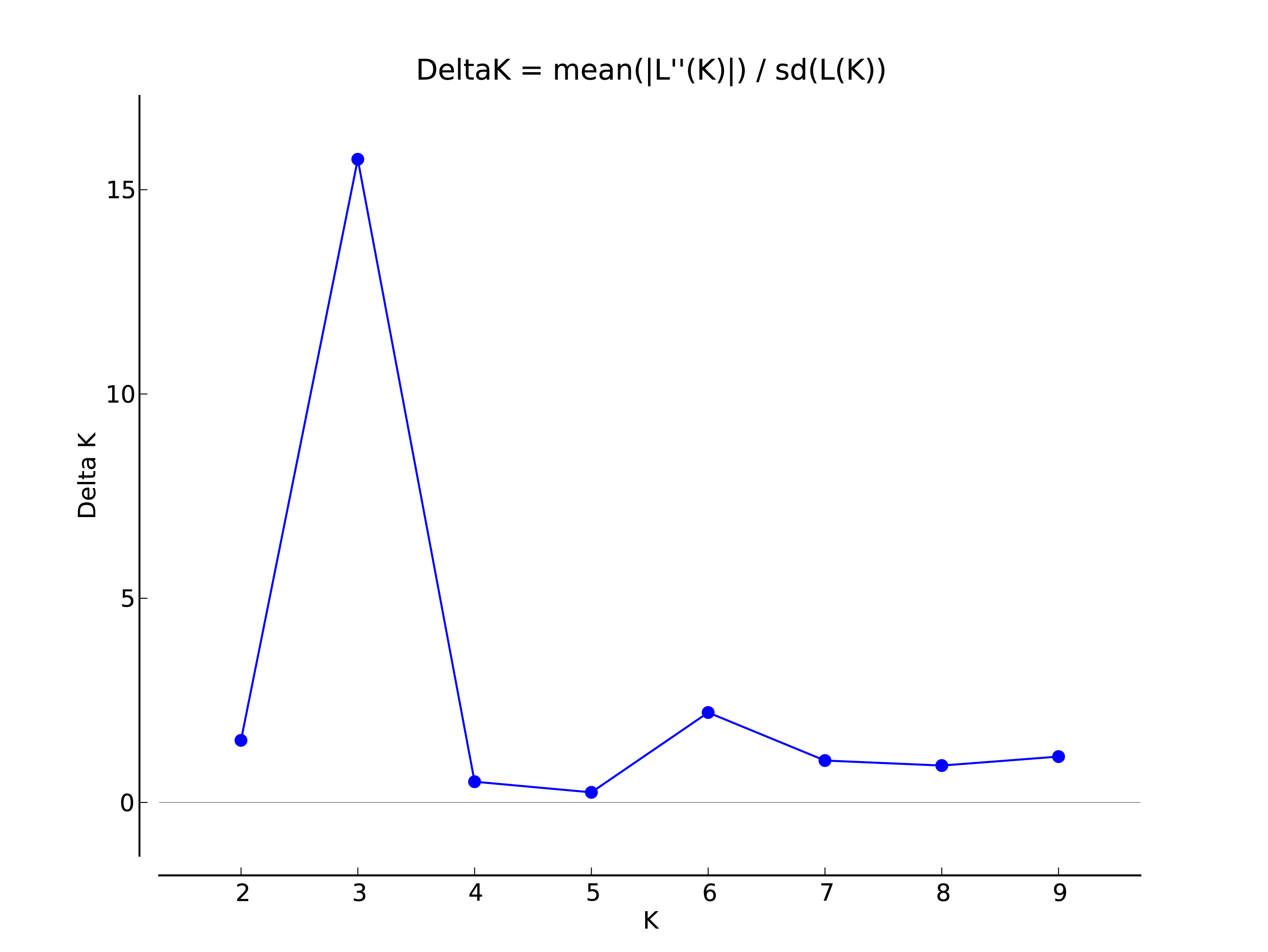

Supplement: S1 Fig — (TIF) [file pone.0249752.s001.tif]
