# Supplementary material for: Genetic diversity and structure of Saussurea polylepis (Asteraceae) on continental islands of Korea: Implications for conservation strategies and management
Source: PLoS One. 2021 Apr 8;16(4):e0249752. doi: 10.1371/journal.pone.0249752 (PMC8031399; doi:10.1371/journal.pone.0249752)
Supplement: S1 Table — (DOCX) [file pone.0249752.s002.docx]

S1 Table. Characteristics of 19 microsatellite markers used in this study.

| Locus | Primer sequences (5'-3') | Repeat motif | Fluorescent dye | GenBank accession no. |
| --- | --- | --- | --- | --- |
| SP01 | F: TCACAAGCCCATTCAGCCAT R: TACAGGCGACCCAAAGTGTC | (CT)_10_ | FAM | MK285611 |
| P02 | F: CAAGGCCGGAGCTTTTCGTA R: TCCATTCGCACCGCTTCTTA | (TC)_11_ | HEX | MK285612 |
| SP03 | F: CTGCCGGAGTCTGCTTCTAG R: TTTGGTCTCACACCCCTTGG | (AAT)_10_ | FAM | MK285613 |
| SP04 | F: CCGAACCCGTACTACAACCC R: TGCCAAGAGATCAACTAGGACG | (AGT)_5_ | HEX | MK285614 |
| SP06 | F: CTCAAGGAGTGGAAACCCCC R: AAAAGGTCGGGGTCTCAACC | (AC)_10_ | FAM | MK285615 |
| SP07 | F: CTCTGCAAGGATCGCTCCAA R: CAATCGAGGCCCTAACCGAA | (GA)_11_ | FAM | MK285616 |
| SP10 | F: GTTTTCCGGCCTCAACCAAC R: AACAATCCGGTAGCTGCCTC | (GGA)_7_ | FAM | MK285610 |
| SP12 | F: AGCGATTCAGATGTTGCCTCT R: ACAACATGTGGTTTCTCATGGT | (TTG)_9_ | FAM | MK285618 |
| SP13 | F: GCATACCGTCCCGATGAAGT  R: CCATCCCATGAATTGCGCAT | (TCTA)_8_ | FAM | MK285619 |
| SP20 | F: GCTTCTCTACTCTTCGCCACA R: GTTTTCAGGGCCAGCCTTTG | (TAG)_7_ | FAM | MK285621 |
| SP21 | F: TGCCTCAACCTGATCGATCG R: TGACCAACCTTGTGCCAGAA | (TCT)_8_ | FAM | MK285622 |
| SP22 | F: TCAACAACCCCGACGAACAT R: CCTCTCATTTTGTGCACCATGG | (TGTT)_6_ | HEX | MK285623 |
| SP23 | F: ACCAGATTGTAGCACCCTCA R: CGCACATTTGGAGATAGCCG | (ATAC)_7_ | HEX | MK285624 |
| SP25 | F: GAACAACAAGGTTTTCCGGCA  R: GCCTCAGTTTCTCATGCCTA | (ATAC)_7_ | FAM | MK285625 |
| SP26 | F: GCCCTAGCCTTTCTTGATGGA R: CGACTGCGCTACATAGGGTT | (GC)_6_ | FAM | MK285626 |
| SP29 | F: TGTCGCGCAAGTCTTCATCT R: CAATAGCCGACTGACTCGCT | (GTT)_7_ | HEX | MK285627 |
| SP31 | F: ACTGAATCGCTGCTTCTGCT R: TGTCCATTCATGCTCTTTGTCA | (ATAG)_8_ | FAM | MK285628 |
| SP34 | F: TGCTAGCAACAACGACTTGT R: GATGTACCGCACTGCATTGC | (TAAA)_6_ | HEX | MK285629 |
| SP35 | F: TTGGTGGAAGGCATGATGGA  R: TCCATGGATGTTTTTGCATTGGT | (CATA)_10_ | FAM | MK285630 |
