# Supplementary material for: Genetic diversity and structure of Saussurea polylepis (Asteraceae) on continental islands of Korea: Implications for conservation strategies and management
Source: PLoS One. 2021 Apr 8;16(4):e0249752. doi: 10.1371/journal.pone.0249752 (PMC8031399; doi:10.1371/journal.pone.0249752)
Supplement: S2 Table — Na = No. of different alleles, Ne = No. of effective alleles, Ho = Observed heterozygosity, He = Expected heterozygosity, F = Fixation index, Fsta = global Fst without using the ENA correction, Fstb = global Fst using the ENA correction, * Significant departures from Hardy-Weinberg equilibrium at p < 0.05. (DOCX) [file pone.0249752.s003.docx]

**S2 Table. Genetic characters of the 19 microsatellite loci.** *Na* = No. of different alleles, *Ne* = No. of effective alleles, *Ho* = Observed heterozygosity, *He* = Expected heterozygosity,

*F* = Fixation index, *Fst^a^* = global *Fst* without using the ENA correction, *Fst^b^* = global *Fst* using the ENA correction, * Significant departures from Hardy-Weinberg equilibrium at *p* < 0.05.

|  | *Na* | *Ne* | *Ho* | *He* | *F* | *Fst^a^* | *Fst^b^* | HWE |
| --- | --- | --- | --- | --- | --- | --- | --- | --- |
| SP1 | 3.20 | 1.88 | 0.45 | 0.40 | -0.13 | 0.067 | 0.067 | - |
| SP2 | 3.80 | 1.98 | 0.58 | 0.47 | -0.24 | 0.037 | 0.036 | - |
| SP3 | 6.40 | 3.26 | 0.24 | 0.56 | 0.60 | 0.111 | 0.100 | * |
| SP4 | 2.80 | 1.39 | 0.35 | 0.23 | -0.33 | 0.107 | 0.106 | - |
| SP6 | 4.80 | 2.69 | 0.58 | 0.52 | -0.13 | 0.183 | 0.183 | - |
| SP7 | 9.60 | 5.56 | 0.88 | 0.78 | -0.20 | 0.066 | 0.068 | - |
| SP10 | 2.80 | 2.55 | 0.72 | 0.60 | -0.23 | 0.023 | 0.0242 | - |
| SP12 | 5.20 | 3.05 | 0.66 | 0.57 | -0.23 | 0.125 | 0.127 | - |
| SP13 | 4.40 | 3.41 | 0.20 | 0.60 | 0.66 | 0.045 | 0.040 | * |
| SP20 | 1.80 | 1.23 | 0.23 | 0.13 | -0.36 | 0.223 | 0.221 | - |
| SP21 | 1.60 | 1.04 | 0.04 | 0.03 | -0.04 | -0.003 | -0.004 | - |
| SP22 | 2.20 | 1.50 | 0.18 | 0.28 | 0.28 | 0.040 | 0.066 | * |
| SP23 | 7.40 | 4.12 | 0.42 | 0.63 | 0.32 | 0.097 | 0.104 | * |
| SP25 | 3.60 | 2.24 | 0.65 | 0.55 | -0.20 | 0.071 | 0.068 | - |
| SP26 | 1.80 | 1.22 | 0.05 | 0.17 | 0.67 | -0.016 | 0.011 | * |
| SP29 | 3.60 | 2.37 | 0.53 | 0.48 | -0.07 | 0.070 | 0.066 | - |
| SP31 | 4.00 | 1.33 | 0.25 | 0.22 | -0.08 | 0.171 | 0.165 | - |
| SP34 | 3.00 | 1.76 | 0.41 | 0.41 | 0.04 | 0.062 | 0.053 | - |
| SP35 | 5.20 | 3.08 | 0.56 | 0.61 | 0.04 | 0.081 | 0.068 | * |
| Mean | 4.06 | 2.40 | 0.42 | 0.43 | 0.01 | 0.084 | 0.083 |  |
