# Supplementary material for: Genetic diversity and structure of Saussurea polylepis (Asteraceae) on continental islands of Korea: Implications for conservation strategies and management
Source: PLoS One. 2021 Apr 8;16(4):e0249752. doi: 10.1371/journal.pone.0249752 (PMC8031399; doi:10.1371/journal.pone.0249752)
Supplement: S3 Table — (DOCX) [file pone.0249752.s004.docx]

**S3 Table. Pairwise genetic differentiation index (*Fst*) (below the diagonal) and Nei’s genetic distance (above the diagonal) among the five populations.**

| Population | GM | GG | HS | HO | UI |
| --- | --- | --- | --- | --- | --- |
| GM | 0.00 | 0.59 | 0.54 | 0.62 | 0.55 |
| GG | 0.27 | 0.00 | 0.11 | 0.14 | 0.14 |
| HS | 0.25 | 0.04 | 0.00 | 0.08 | 0.09 |
| HO | 0.31 | 0.06 | 0.03 | 0.00 | 0.11 |
| UI | 0.24 | 0.05 | 0.04 | 0.05 | 0.00 |
