# Supplementary material for: Genetic diversity and structure of Saussurea polylepis (Asteraceae) on continental islands of Korea: Implications for conservation strategies and management
Source: PLoS One. 2021 Apr 8;16(4):e0249752. doi: 10.1371/journal.pone.0249752 (PMC8031399; doi:10.1371/journal.pone.0249752)
Supplement: S4 Table — Note: + = significant linkage disequilibrium (p < 0.05); − = non-significant linkage disequilibrium; * = unavailable data. (DOCX) [file pone.0249752.s005.docx]

**S4 Table. Linkage disequilibrium among 19 primer pairs of five populations.** Note: + = significant linkage disequilibrium (*p* < 0.05); - = non-significant linkage disequilibrium; * = unavailable data.

|  | SP01 | SP02 | SP03 | SP04 | SP06 | SP07 | SP10 | SP12 | SP13 | SP20 | SP21 | SP22 | SP23 | SP25 | SP26 | SP29 | SP31 | SP34 | SP35 |
| --- | --- | --- | --- | --- | --- | --- | --- | --- | --- | --- | --- | --- | --- | --- | --- | --- | --- | --- | --- |
| SP01 | * |  |  |  |  |  |  |  |  |  |  |  |  |  |  |  |  |  |  |
| SP02 | - | * |  |  |  |  |  |  |  |  |  |  |  |  |  |  |  |  |  |
| SP03 | - | - | * |  |  |  |  |  |  |  |  |  |  |  |  |  |  |  |  |
| SP04 | - | - | - | * |  |  |  |  |  |  |  |  |  |  |  |  |  |  |  |
| SP06 | - | **+** | - | - | * |  |  |  |  |  |  |  |  |  |  |  |  |  |  |
| SP07 | + | - | + | - | + | * |  |  |  |  |  |  |  |  |  |  |  |  |  |
| SP10 | - | + | - | - | + | - | * |  |  |  |  |  |  |  |  |  |  |  |  |
| SP12 | + | - | - | - | - | - | - | * |  |  |  |  |  |  |  |  |  |  |  |
| SP13 | - | - | - | - | + | - | - | - | * |  |  |  |  |  |  |  |  |  |  |
| SP20 | - | - | - | - | - | - | - | - | - | * |  |  |  |  |  |  |  |  |  |
| SP21 | - | - | - | - | - | - | - | - | - | - | * |  |  |  |  |  |  |  |  |
| SP22 | - | - | - | - | - | - | - | - | - | - | - | * |  |  |  |  |  |  |  |
| SP23 | - | + | + | - | + | + | - | - | + | - | - | - | * |  |  |  |  |  |  |
| SP25 | - | - | + | - | - | + | - | - | - | - | - | - | + | * |  |  |  |  |  |
| SP26 | - | - | - | - | - | - | + | - | - | - | - | - | - | - | * |  |  |  |  |
| SP29 | - | - | - | + | - | - | - | - | - | - | - | - | - | - | - | * |  |  |  |
| SP31 | - | - | - | - | - | - | - | - | - | - | - | - | - | - | - | - | * |  |  |
| SP34 | + | - | - | - | - | + | - | - | - | - | - | + | - | - | - | - | - | * |  |
| SP35 | + | - | + | - | + | + | - | - | - | - | - | - | - | - | - | - | - | - | * |
